# Disparities in Breast Cancer Diagnosis, Treatment, and Outcomes Among South Asian American Women

**DOI:** 10.3390/cancers18172866

**Published:** 2026-09-04

**Authors:** Jasmin Hundal, Ishan Gupta, Ashiya Loomba, Yanwen Chen, Halle Moore, Sudipto Mukherjee, Abhay Singh

**Affiliations:** 1 Department of Hematology and Medical Oncology, Taussig Cancer Center, Cleveland Clinic, Cleveland, OH 44106, USA; hundalj@ccf.org (J.H.); mooreh1@ccf.org (H.M.); mukhers2@ccf.org (S.M.); 2 Department of Medicine, All India Institute of Medical Sciences (AIIMS), New Delhi 110029, India; ishan17gupta@aiims.edu; 3 Department of Medicine, Maharaja Agrasen Medical College, Hisar 125047, India; loombaa2@ccf.org; 4 Department of Biostatistics and Quantitative Health Science, Cleveland Clinic, Cleveland, OH 44195, USA; cheny4@ccf.org

**Keywords:** breast cancer, South Asian Americans, health disparities

## Abstract

Background: South Asian Americans (SAAs) represent the fastest-growing U.S. immigrant group but remain underrepresented in breast cancer research. This study utilizes the National Cancer Database (NCDB) to evaluate differences in tumor characteristics, treatment patterns, and survival outcomes between SAAs and non-Hispanic Whites (NHWs). Materials and Methods: A retrospective cohort analysis was conducted using NCDB data from 2004–2021. Women with breast cancer were stratified by race/ethnicity (SAA vs. NHW), and demographic, clinical, and treatment variables were compared. Outcomes assessed were overall survival (OS) and treatment delays, defined as initiation of surgery, chemotherapy, or radiation therapy > 60 days after diagnosis. Multivariable Cox proportional hazards models assessed OS. Results: Among 2,363,627 patients, 20,561 (0.9%) were SAAs and 2,343,066 (99.1%) NHWs. SAAs were younger at diagnosis, with 37.6% aged 20–49 vs. 20.6% of NHWs (*p* < 0.001). Insurance coverage differed, with SAAs more likely privately insured (63.0% vs. 54.2%, *p* < 0.001), less likely on Medicare (17.2% vs. 37.9%), and more often uninsured (4.5% vs. 1.2%). Time to first treatment was longer for SAAs (39.55 vs. 37.17 days, *p* < 0.001). Surgical delays >60 days increased mortality by 59%, while chemotherapy delays raised it by 44%. SAAs demonstrated higher survival at 5, 10, and 15 years (93%, 87%, 81%) vs. NHWs (87%, 76%, 64%). Median survival was 225.8 months but not estimable for SAAs. SAAs presented with aggressive subtypes: triple-negative and HER2-positive tumors. Conclusions: SAAs present younger with aggressive subtypes and treatment delays yet maintain survival advantages; reducing care barriers and clarifying tumor biology are vital to improving outcomes.

## Simple Summary

Breast cancer is the most common cancer diagnosed in women, yet certain populations remain underrepresented in research. South Asian Americans, individuals of Indian, Pakistani, Bangladeshi, and related heritage, are the fastest-growing immigrant population in the United States, but how breast cancer affects them is not well understood, in part because they are frequently combined with the broader Asian American category, which can obscure meaningful differences. Using a large national cancer database, we examined nearly 2.4 million women diagnosed between 2004 and 2021 to compare South Asian American women with white women in terms of tumor characteristics, treatment, and survival. We observed that South Asian American women were diagnosed at younger ages, more frequently presented with aggressive forms of the disease, and experienced longer delays before starting treatment. Despite these factors, they demonstrated more favorable long-term survival, although delays before surgery were associated with higher mortality. These findings underscore the importance of further investigating breast cancer risk, treatment access, and outcomes among South Asian American women. The younger age at diagnosis, higher frequency of aggressive tumor subtypes, and longer time to treatment observed in this population suggest potential opportunities to refine screening approaches and reduce barriers to timely care. Understanding the biological, clinical, and sociodemographic factors underlying their favorable survival outcomes may help inform more equitable and culturally responsive strategies for breast cancer prevention, diagnosis, and treatment.

## 1. Implications for Practice

South Asian Americans (SAAs) present a unique epidemiology of breast cancer, with being diagnosed at younger ages and presenting with more aggressive breast cancer subtypes. Data also show that SAAs experience treatment delays, highlighting the need to design culturally aligned care pathways, including earlier screening efforts, particularly for younger SAA women who do not meet current age-based screening thresholds, as well as reducing delays in surgery and chemotherapy. Although survival outcomes remain favorable, focused interventions addressing insurance gaps and access to healthcare, as well as biological differences, could further improve timely access to care and long-term clinical outcomes in SAAs.

## 2. Introduction

Breast cancer is the most common malignancy among women, with approximately 2.3 million new cases diagnosed worldwide each year [1], and it accounts for nearly 31% of the new cancer cases in women [2]. Breast cancer development is influenced by a combination of genetic, hormonal, reproductive, and lifestyle-related factors, including obesity, physical inactivity, and metabolic dysfunction [3]. Emerging evidence also highlights the role of adipocytokines and chronic low-grade inflammation associated with excess adiposity in promoting tumor initiation and progression [3]. Despite substantial advances in screening and treatment, disparities in breast cancer presentation, management, and outcomes persist across racial and ethnic groups. Understanding these differences is essential to ensuring equitable cancer care and identifying populations that may benefit from tailored prevention, screening, and treatment strategies.

South Asian Americans (SAAs), individuals with ancestry from India, Pakistan, Sri Lanka, Bangladesh, Nepal, and Bhutan, represent one of the fastest immigrant populations in the United States. A major challenge is that SAAs are frequently grouped within the broader Asian American category, which encompasses numerous ethnically and culturally diverse populations with distinct genetic backgrounds, migration histories, socioeconomic circumstances, and health outcomes [1,4,5,6,7,8,9,10]. Aggregating these groups may mask important differences in breast cancer characteristics and outcomes and limit the ability to identify population-specific disparities.

The existing literature on breast cancer among SAAs is limited and has largely been derived from studies of South Asian women outside the United States or from analyses that combine SAAs with other Asian subgroups. Prior studies have suggested that South Asian women may be diagnosed at younger ages and present with less favorable tumor characteristics compared with other populations. However, findings regarding treatment patterns and survival have been inconsistent, and few large-scale studies have specifically examined breast cancer outcomes among SAAs in the United States [11,12,13]. Furthermore, little is known about whether SAAs experience differences in treatment utilization or delays in care, both of which may influence long-term outcomes.

Given the rapid growth of the South Asian American population and the limited availability of population-specific data, a better understanding of breast cancer presentation, treatment, and survival in this group is needed. Therefore, we used the National Cancer Database (NCDB) to compare demographic characteristics, tumor features, stage at diagnosis, treatment patterns, treatment timeliness, and overall survival between South Asian American and non-Hispanic White women diagnosed with breast cancer. By disaggregating SAAs from the broader Asian American category, this study aims to address an important gap in the literature and provide evidence to inform more equitable and culturally responsive breast cancer care.

## 3. Materials and Methods

### 3.1. Study Design and Data Source

This retrospective cohort study utilized data from the National Cancer Database (NCDB), which collects cancer-related data from over 1500 Commission on Cancer (CoC)-accredited facilities across the United States [14]. The NCDB captures approximately 70% of all newly diagnosed cancers in the U.S. and includes demographic, clinical, treatment, and survival data.

### 3.2. Study Population and Inclusion Criteria

The study included women aged 18 years or older who were diagnosed with breast cancer between 2004 and 2021. Breast cancer diagnoses were classified according to the International Classification of Diseases for Oncology, Third Edition (ICD-O-3). Race and ethnicity were self-reported, with South Asian American (SAA) patients identified using NCDB codes “15” (Asian Indian or Pakistani, NOS), “16” (Asian Indian), and “17” (Pakistani). Non-Hispanic White (NHW) patients were defined as those with race code “01” and Hispanic ethnicity coded as 0.

Patients younger than 18 years, male patients, individuals with races or ethnicities other than the predefined SAA and NHW cohorts, and patients with missing race or ethnicity information were excluded. Patients diagnosed outside the study period (2004–2021) were also excluded.

### 3.3. Variables and Outcomes

The primary exposure variable was race/ethnicity (SAA vs. NHW). Additional demographic, clinical, and socioeconomic variables included age at diagnosis, tumor characteristics such as histology, grade, and receptor subtype, stage at presentation, comorbidities assessed using the Charlson–Deyo Comorbidity Index, socioeconomic indicators such as median household income and percentage of adults without a high school diploma, insurance status, treatment modalities including receipt and timing of surgery, chemotherapy, radiation therapy, and hormone therapy, and facility type categorized as an academic/research center versus a community hospital.

The primary outcome of interest was overall survival (OS), defined as the time from diagnosis to death or last follow-up. Secondary outcomes included treatment delays, measured as time to surgery, chemotherapy, or radiation therapy initiation beyond 60 days from diagnosis.

### 3.4. Tumor Subtype Classification

For patients diagnosed in or after 2010, molecular subtypes were defined using hormone receptor (HR) and HER2 status. HR-positive tumors included those with ER and/or PR positivity, while HR-negative tumors were ER- and PR-negative. Subtypes were categorized as HR+/HER2−, HER2+ (any HR), and triple-negative breast cancer (TNBC: ER−, PR−, HER2−).

### 3.5. Statistical Analysis

Descriptive statistics were used to compare patient characteristics between SAAs and NHWs. Chi-square tests were used for categorical variables, while continuous variables were compared using *t*-tests.

OS was defined as the time from diagnosis to death or last follow-up. Cox proportional hazards models were used to estimate the effect of race/ethnicity on OS unadjusted, or after adjusted for age group, insurance status, stage at diagnosis, Charlson–Deyo comorbidity score, socioeconomic indicators (percent without a high school degree and median income quartiles), facility type, urban or rural residence, treatment types (chemotherapy, radiation, surgery, hormone therapy) and tumor grade. We reported hazard ratios and 95% CIs. R package tableone and survival were used for the demographic table, the OS table, and the comparison. All analyses were conducted via SAS 9.4, or R 4.2.2. A *p*-value of <0.05 was considered statistically significant.

### 3.6. Ethical Considerations

This study used de-identified NCDB data adhering to ethical standards for patient privacy and confidentiality.

## 4. Results

### 4.1. General Characteristics

We analyzed data on 2,363,627 patients with breast cancer, of which 2,343,066 (99.1%) were NHW and 20,561 (0.9%) were SAA. The mean age at diagnosis was 61.07 years, with SAAs being significantly younger at 54.85 years compared to NHWs at 61.13 years. All patients in this cohort were female, with no males. Age distribution varied significantly, with a higher proportion of SAAs diagnosed at younger ages. Among SAAs, 37.6% were between the ages of 20–49 years, compared to 20.6% of NHWs (*p* < 0.001). In contrast, SAAs had a smaller proportion in older age groups, including 65–74 years (18.0% vs. 24.5%) and 75+ years (6.7% vs. 16.4%), (*p* < 0.001).

The distribution of diagnoses over time varied, with SAAs having a higher proportion of diagnoses in more recent years (Table 1). Using the total number of SAA or NHW breast cancer cases as the denominator and the number of cases diagnosed during each period as the numerator, we observed that the proportion of diagnoses among SAAs increased in more recent years. Between 2014 and 2018, 36.0% of SAAs were diagnosed compared to 30.8% of NHWs, and between 2019 and 2021, 26.5% of SAAs were diagnosed compared to 18.7% of NHWs (*p* < 0.001). Regarding insurance status, SAAs were more likely to be privately insured (63.0% vs. 54.2%, *p* < 0.001) and had a lower proportion covered by Medicare (17.2% vs. 37.9%, *p* < 0.001). Additionally, SAAs had a higher proportion of uninsured patients (4.5% vs. 1.2%, *p* < 0.001).

Geographic and socioeconomic factors revealed that SAAs were more likely to reside in metropolitan areas (94.1% vs. 83.1%, *p* < 0.001). They were also more likely to live in neighborhoods with a higher median income, with 62.2% of SAAs residing in areas where the median income was ≥$74,063 compared to 39.3% of NHWs. Furthermore, SAAs were more likely to seek care at academic or research institutions (39.3% vs. 25.6%, *p* < 0.001), whereas NHWs were more likely to receive care at comprehensive community cancer programs (41.8% vs. 30.0%, *p* < 0.001).

Stage distribution also showed differences, with a higher proportion of SAAs diagnosed at Stage 0 (19.3% vs. 17.7%, *p* < 0.001) and Stage II (24.0% vs. 21.5%, *p* < 0.001) (Table 1, Supplementary Table S1). However, the proportion of Stage IV cases was similar between the two groups (3.9% vs. 3.8%, *p* < 0.001). The burden of comorbid conditions was lower among SAAs, with 86.2% having a Charlson–Deyo Comorbidity Score of 0 compared to 85.1% of NHWs (*p* < 0.001).

### 4.2. Treatment Differences

Treatment patterns varied significantly between SAA and NHW patients with breast cancer (Supplementary Table S2). The time to first treatment differed significantly between SAAs and NHWs, with a median time of 37.17 days among NHWs compared to 39.55 days among SAAs (*p* < 0.001).

### 4.3. Survival Outcomes

Survival outcomes demonstrated notable differences between SAA and NHW breast cancer patients across all stages of the disease. Overall, SAAs had superior survival rates compared to NHWs. The 5-year survival rate for SAAs was 93% (95% CI: 92–93%) compared to 87% (95% CI: 87–87%) for NHWs. This trend persisted at 10 years (87% vs. 76%) and 15 years (81% vs. 64%). The median survival time for all patients was 225.81 months. However, it was not estimable for SAAs due to their more favorable survival. Stage-specific survival analyses revealed that SAAs demonstrated superior survival rates across all stages of breast cancer at 1, 3, and 5 years (Supplementary Tables S3 and S5).

In multivariable analysis, SAA patients had a 45% lower mortality risk than NHWs after adjusting for demographic, socioeconomic, clinical, and treatment factors (Table 2, Supplemental Table S4). Old age, Medicaid coverage, and lower income were associated with worse survival, while private insurance (0.61, 95% CI 0.58–0.63, *p* < 0.0001) and treatment at an academic center (*HR:* 0.81, 95% CI 0.80–0.82, *p* < 0.0001) were associated with improved survival. Delays in surgery beyond 60 days were associated with a 30% increase in mortality (*p* < 0.0001).

To better understand factors associated with survival within the SAA population, a multivariable analysis was conducted exclusively among SAA breast cancer patients to identify clinical and sociodemographic predictors of mortality (Supplemental Table S5). Several clinical and sociodemographic factors were found to impact survival outcomes significantly. Increasing age was strongly associated with a higher mortality risk. Relative to women aged 20–49 years, the hazard of death rose by 22% (HR 1.22, 95% CI 1.07–1.39; *p* = 0.003) in those aged 50–64, by 60% (HR 1.60, 1.38–1.87; *p* < 0.0001) in those aged 65–74, and more than five-fold (HR 5.02, 4.31–5.84; *p* < 0.0001) in those aged 75 and older.

Insurance status was also significantly associated with survival. Relative to uninsured patients, those with private insurance showed a 62% reduction in mortality risk (HR 0.38, 95% CI 0.32–0.46; *p* < 0.0001). Government insurance, Medicaid and Medicare did not show a significant survival advantage. Socioeconomic status, as measured by median income, did not significantly impact survival outcomes in this population.

Disease stage at diagnosis remained the most significant predictor of survival.

Relative to stage I, mortality increased progressively with disease stage: stage II, HR 1.97 (95% CI, 1.71–2.28); stage III, HR 4.69 (4.03–5.47); stage IV, HR 19.96 (17.14–23.25). Histologic grade showed a stepwise association with mortality. (Supplementary Table S5).

In South Asian American women, triple-negative breast cancer was associated with a 74% higher mortality hazard (HR 1.74, 95% CI 1.30–2.32). Mortality increased with higher Charlson–Deyo scores: compared with individuals without comorbidities, a score of 1 raised the hazard by 42% (HR 1.42, 95% CI 1.23–1.64), a score of 2 more than doubled it (HR 2.61, 95% CI 1.94–3.51), and a score of 3 or greater nearly quadrupled the risk (HR 4.21, 95% CI 2.85–6.21) (Supplementary Table S5).

## 5. Discussion

South Asian Americans are the fastest-growing ethnic minority in the United States. The study consisted of 2,363,627 patients diagnosed with breast cancer, of which 2,343,066 (99.1%) were NHW and 20,561 were SAA. Our study identifies differences in the presentation, access to treatment, and treatment and survival outcomes between these populations. We found that SAA women were diagnosed at a younger age and had more aggressive subtypes. Despite experiencing delays in treatment initiation, SAA women demonstrated superior long-term overall survival compared to NHW women.

Previous studies have highlighted differences in the incidence of breast cancer among various ethnic and racial groups but with a lower focus on SAAs [15]. Given the distinct genetic predispositions, cultural practices, and lifestyle behaviors, these factors may contribute to different patterns of breast cancer incidence and outcomes. South Asian populations have a higher burden of type 2 diabetes and coronary artery disease, reflecting distinct cardiometabolic risk profiles that may also influence cancer risk, treatment tolerance, and long-term outcomes [16,17,18,19,20]. Additionally, there is an SAA paradox in which SAAs are at a higher risk of coronary artery disease, but a lower risk of cancer and venous thromboembolism compared to other populations [17]. This contrast underscores the need for further research to unravel the mechanisms behind these disparities [17].

In our study, SAA women were diagnosed at a significantly younger age than NHW women, with a mean age of 54.85 years compared to 61.13 years. In the SAA group, 37.6% of women were younger than 50, compared with 20.6% in the NHW group. This finding aligns with a previous study from England by Gathani et al., which also highlighted that South Asian women were diagnosed with BC at younger ages as compared to White women [21]. More recent years showed a higher proportion of diagnoses, though this pattern is also consistent with the growth of SAA populations over the study period and cannot be distinguished from a true change in incidence in the data. Future studies are needed to determine the actual incidence patterns, detection rates, and potential screening gaps in this population.

Tumor biology differed between the groups, which may have contributed to their higher rates of chemotherapy use. South Asian women had more HER2-positive disease (12.0% vs. 8.3%) and triple-negative breast cancer (8.0% vs. 6.0%), reflecting a greater burden of aggressive subtypes (Table 1). Despite higher rates of aggressive subtypes, SAA women had a superior long-term overall survival rate compared to NHWs. This aligns with prior studies, including one that highlighted the survival advantage of South Asians in England, which has been attributed in part to better socioeconomic status and access to high-quality healthcare [15,21,22]. Our study suggests that South Asian ethnicity is independently associated with better overall survival outcomes and a lower risk of mortality even after adjusting for socioeconomic status and clinical or treatment factors. The superior overall survival rates among SAAs have also been noted in colorectal cancer [22].

The finding of younger age at diagnosis, a greater proportion of aggressive tumor subtypes, and treatment delays despite superior overall survival among SAA women is unlikely to be explained by a single factor. These findings may be considered within an intersectional framework, recognizing that breast cancer outcomes are shaped by the interaction of clinical characteristics with social and healthcare factors [23,24]. In our cohort, SAA women were more likely to receive care at academic or research centers, had higher rates of private insurance, and more commonly lived in higher-income neighborhoods. These factors may have influenced access to specialty care and treatment and could partly explain the favorable survival observed despite differences in tumor characteristics and treatment timing.

At the same time, SAA women also had a higher proportion of uninsured patients and experienced a longer time to treatment initiation. These findings highlight heterogeneity within the SAA population and suggest that the observed survival advantage should not be interpreted as uniformly favorable access to care. Rather, the survival differences observed in this study likely reflect the combined influence of tumor characteristics, socioeconomic factors, and healthcare access.

The NCDB does not capture several factors that may further shape these relationships, including immigration history, language proficiency, health literacy, individual-level socioeconomic status, and cultural factors. These limitations prevent direct evaluation of how these factors interact with treatment delivery and clinical outcomes. Future studies incorporating individual-level social determinants and more detailed clinical data are needed to better understand the factors contributing to breast cancer outcomes among SAA women.

Although our analysis showed higher overall survival in these populations than NHWs, examining survival within the SAA group alone revealed that disease stage at diagnosis significantly affected outcomes. Compared to Stage I breast cancer, Stage II was associated with nearly double the mortality risk. For Stage III disease, the risk increased almost fivefold. Patients diagnosed with Stage IV disease faced the highest risk, with nearly a 20-fold increase in mortality. Tumor grade also significantly influenced survival, as poorly differentiated tumors carried a 2.5-fold increased mortality risk. These associations may reflect underlying molecular distinctions in the tumors of South Asian American patients, but additional studies are needed to confirm this.

Our findings highlight the significant influence of disease stage, tumor biology, and comorbid conditions on overall survival outcomes among SAA women. Notably, SAAs are disproportionately affected by cardiometabolic diseases, including high rates of atherosclerotic cardiovascular disease, diabetes, and metabolic syndrome. In our analysis, older age was associated with a higher mortality risk, with patients aged 50–64 at moderately increased risk, while those aged 65–74 faced significantly higher risk. The highest mortality was observed in patients aged 75 and older, who experienced a fivefold increase in risk. Because NCDB does not capture cause of death, we cannot determine whether these age-related differences reflect cancer-specific mortality or competing causes. However, age-related comorbidities may contribute to poorer cancer outcomes by increasing treatment-related toxicity, reducing endocrine therapy efficacy, and exacerbating cardiovascular complications from chemotherapy and radiation therapy. SAAs’ increased susceptibility to visceral adiposity and insulin resistance may further place them at risk for metabolic dysfunction, which could negatively affect treatment tolerance and long-term survival.

A syndemic framework may provide a useful basis for future investigation of breast cancer outcomes among SAA women, particularly given the high burden of cardiometabolic disease in South Asian populations [25]. Diabetes, cardiovascular disease, visceral adiposity, and insulin resistance may influence treatment tolerance, competing mortality risk, and long-term survivorship outcomes. These conditions may also intersect with social and healthcare factors that shape access to care and treatment delivery. However, the NCDB does not capture the individual-level metabolic, social, or longitudinal data required to formally evaluate these interactions. Therefore, the present findings do not establish a syndemic relationship but support further investigation of how cardiometabolic, clinical, and social factors may jointly influence breast cancer outcomes in SAA women.

### Limitations and Future Research Directions

This study has several limitations, including those inherent to its retrospective design and to use of the National Cancer Database (NCDB). As a strength, the NCDB is one of the largest cancer registries in the world, capturing approximately 70% of newly diagnosed cancers in the United States and providing detailed demographic, clinical, treatment, and survival data that enabled us to assemble a sizable South Asian American (SAA) cohort that would be difficult to study in smaller datasets. Nevertheless, the NCDB includes only patients treated at Commission on Cancer–accredited facilities, which tend to be larger and more often academic, potentially introducing selection bias favoring insured, higher-income SAA patients and underrepresenting recent immigrants, uninsured individuals, and those in rural settings. Socioeconomic variables were also derived from neighborhood-level data rather than individual-level measures, potentially reducing accuracy.

Because our study period spanned 2004 to 2021, several changes in NCDB reporting practices may affect data completeness and comparability across the cohort. Clinical staging became mandatory only in 2008, and the AJCC staging system transitioned from the 6th to the 7th edition in 2010. Standardized hormone receptor and HER2 reporting was not reliably available before 2010, which is why our molecular subtype classification was restricted to patients diagnosed in or after 2010; consequently, subtype data were missing for earlier-diagnosed patients. More broadly, the database lacks key clinical and molecular information necessary to fully assess tumor biology and does not capture the staging investigations performed, potentially leading to stage misclassification.

Importantly, the NCDB records vital status but not cause of death or recurrence, so only overall survival could be calculated, and breast cancer–specific and disease-free survival could not be determined. The superior long-term survival observed among SAA women should therefore be interpreted as all-cause mortality. In addition, we excluded patients with missing values for any covariate in the multivariable model through complete-case analysis without imputation, which may introduce selection bias if data were not missing completely at random. Because the NCDB does not include underlying population denominators, we could not calculate population-based breast cancer incidence rates. Therefore, our findings reflect the distribution of breast cancer cases within Commission on Cancer–accredited facilities rather than population-level incidence.

Finally, the classification of SAAs is limited, excluding several subgroups such as Bangladeshi, Sri Lankan, and Nepali populations, thereby restricting the ability to evaluate subgroup-specific disparities. Unmeasured factors, such as cultural influences, health behaviors, and broader social determinants, may also contribute to the observed disparities.

Future research should prioritize expanding the representation of diverse SAA subgroups in national databases and integrating individual-level socioeconomic, cultural, behavioral, and healthcare access data, including immigration history, language proficiency, health literacy, and neighborhood-level social determinants of health. Combining these data with molecular and genomic profiling may provide a more comprehensive understanding of how biological and social determinants jointly influence breast cancer risk, treatment delivery, and survival. Linking cancer registries with incidence and mortality data may further improve understanding of population-level disparities. Investigations into treatment delays, adherence, and survivorship outcomes will help inform evidence-based care strategies. From a policy standpoint, efforts should focus on culturally tailored screening, patient navigation, and survivorship interventions that address barriers across the cancer care continuum.

## 6. Conclusions

In summary, this retrospective analysis of 2,363,627 women with breast cancer demonstrated that South Asian Americans, the fastest-growing ethnic minority in the United States, are diagnosed at younger ages and with more aggressive tumor subtypes, yet demonstrate superior long-term survival compared with non-Hispanic White women, even after adjusting for demographic, socioeconomic, clinical, and treatment factors. This survival advantage persisted despite delays in treatment initiation and likely reflects a combination of population-specific tumor biology that may enhance chemosensitivity and preferential treatment at academic centers offering multidisciplinary, guideline-concordant care. The observed treatment delays and insurance gaps underscore the need for culturally tailored interventions, including earlier screening for younger SAA women who fall below current age-based screening thresholds and reduced barriers to timely, guideline-concordant care. Given the disproportionate burden of cardiometabolic disease in this population, addressing the intersection of metabolic and cancer-related risk factors and integrating cardiometabolic management into cancer care represents an important opportunity to further improve outcomes. Future molecular and genomic studies, alongside expanded representation of diverse SAA subgroups in national databases, are warranted to clarify tumor biology and optimize care in this underrepresented and rapidly growing population.

## Figures and Tables

**Table 1:** Demographic, Clinical, and Treatment Characteristics by Race/Ethnicity.

Characteristic	South Asian (*n* = 20,561)	NHW (*n* = 2,343,066)	*p*-Value
**Age, mean (SD)**	54.85 (12.71)	61.13 (13.04)	<0.001
**Age group**			<0.001
20–49	7741 (37.6%)	482,655 (20.6%)	
50–64	7737 (37.6%)	900,370 (38.4%)	
65–74	3711 (18.0%)	574,752 (24.5%)	
75+	1372 (6.7%)	385,289 (16.4%)	
**Annual New Cases per year**			
2004–2008	2994 (14.4%)	635,077 (24.1%)	<0.001
2009–2013	4794 (23.1%)	716,507 (27.2%)	
2014–2019	7484 (36.0%)	798,868 (30.3%)	
2019–2021	5496 (26.5%)	487,706 (18.5%)	
**Stage at Diagnosis**			<0.001
Stage 0	3975 (19.3%)	414,486 (17.7%)	
Stage I	8237 (40.1%)	1,083,034 (46.2%)	
Stage II	4929 (24.0%)	503,306 (21.5%)	
Stage III	1823 (8.9%)	173,558 (7.4%)	
Stage IV	793 (3.9%)	89,572 (3.8%)	
**Subtype**			<0.001
HR+/HER2−	9225 (44.9%)	1,034,370 (44.1%)	
HR+/HER2+	1653 (8.0%)	137,852 (5.9%)	
HR−/HER2+	828 (4.0%)	56,862 (2.4%)	
Triple Negative	1636 (8.0%)	141,535 (6.0%)	
**Charlson** **–** **Deyo Comorbidity Score**			<0.001
0	17,725 (86.2%)	1,994,157 (85.1%)	
1	2392 (11.6%)	264,763 (11.3%)	
2	305 (1.5%)	57,972 (2.5%)	
≥3	139 (0.7%)	26,174 (1.1%)	
**Chemotherapy**			<0.001
Yes	4999 (24.3%)	391,117 (16.7%)	
No	15,246 (74.2%)	1,920,220 (82.0%)	
**Radiation**			
Yes	11,376 (55.3%)	1,321,791 (56.4%)	
No	8450 (41.1%)	947,800 (40.5%)	
**Surgery**			
Yes	18,863 (91.7%)	2,177,529 (92.9%)	
No	1417 (6.9%)	145,149 (6.2%)	
**Hormonal Therapy**			<0.001
Yes	12,482 (60.7%)	1,448,043 (61.8%)	
No	7287 (35.4%)	823,407 (35.1%)	

**Table 2:** Multivariable Cox Proportional Hazards Regression Analysis of Overall Survival in Breast Cancer Patients.

Variable	Hazard Ratio	95% CI Lower	95% CI Upper	*p*-Value
**Race**				
NHW	ref			
South Asian	0.5452	0.5028	0.5912	<0.0001
**Age group**				
20–49	ref			
50–64	1.4171	1.3875	1.4474	<0.0001
65–74	1.9117	1.8637	1.9609	<0.0001
75+	4.4788	4.3642	4.5963	<0.0001
**Insurance status**				
Not Insured	ref			
Private Insurance	0.6018	0.5778	0.6267	<0.0001
Medicaid	0.996	0.9523	1.0417	0.861876
Medicare	0.8291	0.7948	0.8648	<0.0001
**Median income quartiles**				
≥17.6%	ref			
10.9–17.5%	0.9429	0.9267	0.9593	<0.0001
6.3–10.8%	0.869	0.854	0.8842	<0.0001
<6.3%	0.7555	0.7428	0.7685	<0.0001
**R** **esidence**				
Metro	ref			
Rural	1.0163	0.9782	1.0559	0.408159
Urban	0.9935	0.9781	1.0091	0.40931
**Stage**				
Stage I	ref			
Stage II	1.7467	1.7236	1.7701	<0.0001
Stage III	3.7625	3.6993	3.8268	<0.0001
Stage IV	7.0047	6.8499	7.1629	<0.0001
**Grade**				
1	ref			
2	1.1695	1.1516	1.1876	<0.0001
3	1.5692	1.5419	1.5969	<0.0001
4	1.569	1.4198	1.7339	<0.0001
**Subtype**				
HR−/HER2+	ref			
HR+/HER2−	1.4372	1.3975	1.4781	<0.0001
HR+/HER2+	1.2603	1.2222	1.2997	<0.0001
Triple Negative	1.645	1.6005	1.6907	<0.0001
**Charlson–Deyo Comorbidity Index**				
0	ref			
1	1.4293	1.4099	1.449	<0.0001
2	1.9544	1.9098	2.0001	<0.0001
≥3	2.5829	2.5002	2.6684	<0.0001
**Chemotherapy**				
No	ref			
Yes	**1.0329**	**1.0156**	**1.0506**	<0.0001
**Radiation**				
No	ref			
Yes	0.6945	0.6567	0.7345	<0.0001
**Surgery**				
No	ref			
Yes	0.4318	0.413	0.4513	<0.0001
**Hormone**				
No	ref			
Yes	0.6256	0.6169	0.6344	<0.0001
**Delay in surgery > 60 Days**				
No	ref			
Yes	1.2982	1.2792	1.3175	<0.0001
**Delay in radiation > 60 Days**				
No	ref			
Yes	0.8913	0.8731	0.91	<0.0001
**Delay in chemotherapy > 60 Days**				
No	ref			
Yes	0.8651	0.8499	0.8806	<0.0001
**Facility type**				
Community Cancer Program	ref			
Comprehensive Community Cancer Program	0.9141	0.8976	0.9309	<0.0001
Academic/Research Program	0.8081	0.7921	0.8243	<0.0001
Integrated Network Cancer Program	0.8864	0.8686	0.9045	<0.0001

## Data Availability

Data is available as Supplementary files and on request.

## Supplementary Materials

The following supporting information can be downloaded at: https://www.mdpi.com/article/10.3390/cancers18172866/s1, Table S1: Demographic, Clinical, and Treatment Characteristics by Race/Ethnicity; Table S2. Treatment Receipt, Delays, and Surgical Characteristics Stratified by Stage at Diagnosis; Table S3. Breast Cancer Survival Outcomes by Stage and Ethnicity (Non-Hispanic White vs. South Asian Patients); Table S4: Multivariable Cox Proportional Hazards Regression Analysis of Overall Survival in Breast Cancer Patients; Table S5: Multivariable Cox Proportional Hazards Regression for Overall Survival Among South Asian Breast Cancer Patients.

## Abbreviations

The following abbreviations are used in this manuscript:

AJCC	American Joint Committee on Cancer
ASCVD	Atherosclerotic cardiovascular disease
BC	Breast cancer
CI	Confidence interval
CoC	Commission on Cancer
ER	Estrogen receptor
HER2	Human epidermal growth factor receptor 2
HR	Hazard ratio
ICD-O-3	International Classification of Diseases for Oncology, Third Edition
NCDB	National Cancer Database
NHWs	Non-Hispanic Whites
NOS	Not otherwise specified
OS	Overall survival
PR	Progesterone receptor
SAAs	South Asian Americans
TNBC	Triple-negative breast cancer
