# Supplementary material for: Disparities in Breast Cancer Diagnosis, Treatment, and Outcomes Among South Asian American Women
Source: Cancers (Basel). 2026 Sep 4;18(17):2866. doi: 10.3390/cancers18172866 (PMC13564886; doi:10.3390/cancers18172866)
Supplement: Supplementary file 1 [file cancers-18-02866-s001.zip › cancers-4331787-supplementary.pdf]

Supplementary Table S1: Demographic, Clinical, and Treatment Characteristics by Race/Ethnicity

		Overall	South Asian	NHW	p-value
N		2363627	20561	2343066	
Age		61.07 (13.05)	54.85 (12.71)	61.13 (13.04)	<0.001
Age group	20-49	490396 (20.7)	7741 (37.6)	482655 (20.6)	<0.001
	50-64	908107 (38.4)	7737 (37.6)	900370 (38.4)	
	65-74	578463 (24.5)	3711 (18.0)	574752 (24.5)	
	75+	386661 (16.4)	1372 (6.7)	385289 (16.4)	
Year of Diagnosis	2004-2008	546530 (23.1)	2962 (14.4)	543568 (23.2)	<0.001
	2009-2013	642766 (27.2)	4751 (23.1)	638015 (27.2)	
	2014-2018	730139 (30.9)	7404 (36.0)	722735 (30.8)	
	2019-2021	444192 (18.8)	5444 (26.5)	438748 (18.7)	
Type of Insurance	Not Insured	29092 (1.2)	927 (4.5)	28165 (1.2)	<0.001
	Private Insurance	1282659 (54.3)	12955 (63.0)	1269704 (54.2)	
	Medicaid	101996 (4.3)	2506 (12.2)	99490 (4.2)	
	Medicare	891966 (37.7)	3538 (17.2)	888428 (37.9)	
	Other Government	23624 (1.0)	132 (0.6)	23492 (1.0)	
	Insurance Status Unknown	34290 (1.5)	503 (2.4)	33787 (1.4)	
High school degree quartiles (%)	Unknown	287202 (12.2)	2151 (10.5)	285051 (12.2)	<0.001

	>= 17.6%	269680 (11.4)	2951 (14.4)	266729 (11.4)	
	10.9-17.5%	479048 (20.3)	3229 (15.7)	475819 (20.3)	
	6.3-10.8%	636560 (26.9)	5191 (25.2)	631369 (26.9)	
	< 6.3%	691137 (29.2)	7039 (34.2)	684098 (29.2)	
Residence	Unknown	65776 (2.8)	852 (4.1)	64924 (2.8)	<0.001
	Metro	1967298 (83.2)	19356 (94.1)	1947942 (83.1)	
	Rural	36771 (1.6)	57 (0.3)	36714 (1.6)	
	Urban	293782 (12.4)	296 (1.4)	293486 (12.5)	
Facility Location	Unknown	102483 (4.3)	2209 (10.7)	100274 (4.3)	<0.001
	New England	163535 (6.9)	840 (4.1)	162695 (6.9)	
	Middle Atlantic	343825 (14.5)	6339 (30.8)	337486 (14.4)	
	South Atlantic	475491 (20.1)	3444 (16.8)	472047 (20.1)	
	Central Total	898731 (38.0)	4623 (22.5)	894108 (38.2)	
	Mountain	117217 (5.0)	221 (1.1)	116996 (5.0)	
	Pacific	262345 (11.1)	2885 (14.0)	259460 (11.1)	
Median income quartiles	Unknown	292600 (12.4)	2159 (10.5)	290441 (12.4)	<0.001
	< $46,277	224837 (9.5)	936 (4.6)	223901 (9.6)	
	$46,227-$57,856	409148 (17.3)	1592 (7.7)	407556 (17.4)	
	$57,857-$74,062	503464 (21.3)	3091 (15.0)	500373 (21.4)	

	>= $74,063	933578 (39.5)	12783 (62.2)	920795 (39.3)	
Facility type	Unknown	102483 (4.3)	2209 (10.7)	100274 (4.3)	<0.001
	Community Cancer Program	177507 (7.5)	1050 (5.1)	176457 (7.5)	
	Comprehensive Community Cancer Program	984711 (41.7)	6169 (30.0)	978542 (41.8)	
	Academic/Research Program	607967 (25.7)	8089 (39.3)	599878 (25.6)	
	Integrated Network Cancer Program	490959 (20.8)	3044 (14.8)	487915 (20.8)	
Stage	Stage 0	418461 (17.7)	3975 (19.3)	414486 (17.7)	<0.001
	Stage I	1091271 (46.2)	8237 (40.1)	1083034 (46.2)	
	Stage II	508235 (21.5)	4929 (24.0)	503306 (21.5)	
	Stage III	175381 (7.4)	1823 (8.9)	173558 (7.4)	
	Stage IV	90365 (3.8)	793 (3.9)	89572 (3.8)	
Charlson-Deyo Comorbidity Index	0	2011882 (85.1)	17725 (86.2)	1994157 (85.1)	<0.001
	1	267155 (11.3)	2392 (11.6)	264763 (11.3)	
	2	58277 (2.5)	305 (1.5)	57972 (2.5)	
	>= 3	26313 (1.1)	139 (0.7)	26174 (1.1)	
Grade	Unknown	595218 (25.2)	7167 (34.9)	588051 (25.1)	<0.001
	1	358336 (15.2)	1892 (9.2)	356444 (15.2)	
	2	697316 (29.5)	5269 (25.6)	692047 (29.5)	
	3	506770 (21.4)	4631 (22.5)	502139 (21.4)	

	4	12899 (0.5)	101 (0.5)	12798 (0.5)	
Histology	Unknown	98401 (4.2)	804 (3.9)	97597 (4.2)	<0.001
	Ductal Carcinoma	1783801 (75.5)	16283 (9.2)	1767518 (75.4)	
	Lobular Carcinoma	469807 (19.9)	3382 (16.4)	466425 (19.9)	
	Other	11618 (0.5)	92 (0.4)	11526 (0.5)	
Subtype (%)	Unknown	979666 (41.4)	7219 (35.1)	972447 (41.5)	<0.001
	HR-/HER2+	57690 (2.4)	828 (4.0)	56862 (2.4)	
	HR+/HER2-	1043595 (44.2)	9225 (44.9)	1034370 (44.1)	
	HR+/HER2+	139505 (5.9)	1653 (8.0)	137852 (5.9)	
	Triple Negative	143171 (6.1)	1636 (8.0)	141535 (6.0)	
Chemotherapy	No Treatment	1935466 (81.9)	15246 (74.2)	1920220 (82.0)	<0.001
	Treatment	396116 (16.8)	4999 (24.3)	391117 (16.7)	
	Unknown	32043 (1.4)	316 (1.5)	31727 (1.4)	
Radiation (%)	No Radiation	956250 (40.5)	8450 (41.1)	947800 (40.5)	<0.001
	Radiation	1333167 (56.4)	11376 (55.3)	1321791 (56.4)	
	Unknown	74210 (3.1)	735 (3.6)	73475 (3.1)	
Surgery	No Surgery	146566 (6.2)	1417 (6.9)	145149 (6.2)	<0.001
	Surgery	2196392 (92.9)	18863 (91.7)	2177529 (92.9)	
	Unknown	20669 (0.9)	281 (1.4)	20388 (0.9)	

Hormonal Treatment	No Treatment	830694 (35.1)	7287 (35.4)	823407 (35.1)	<0.001
	Treatment	1460525 (61.8)	12482 (60.7)	1448043 (61.8)	
	Unknown	72407 (3.1)	792 (3.9)	71615 (3.1)	
30-day mortality		305235 (12.9)	3440 (16.7)	301795 (12.9)	<0.001
	Alive or died > 30 days after surgery	2046501 (86.6)	16970 (82.5)	2029531 (86.6)	
	Died <= 30 days after surgery	1602 (0.1)	2 (0.0)	1600 (0.1)	
	No surgery, or alive cases have < 30 days of follow up	10289 (0.4)	149 (0.7)	10140 (0.4)	
90-day mortality		305235 (12.9)	3440 (16.7)	301795 (12.9)	<0.001
	Alive or died > 90 days after surgery	2036882 (86.2)	16860 (82.0)	2020022 (86.2)	
	Died <= 90 days after surgery	5649 (0.2)	12 (0.1)	5637 (0.2)	
	No surgery, or alive cases have < 90 days of follow up	15861 (0.7)	249 (1.2)	15612 (0.7)	
Hormone therapy at any CoC facility	None	677433 (28.7)	6242 (30.4)	671191 (28.6)	<0.001
	Hormone therapy administered as first course therapy	1460525 (61.8)	12482 (60.7)	1448043 (61.8)	
	Not recommended/administered, contraindicated due to patient risk factors	28229 (1.2)	154 (0.7)	28075 (1.2)	
	Hormone therapy not administered, patient died prior to planned or recommended therapy	2948 (0.1)	7 (0.0)	2941 (0.1)	
	Recommended but not administered, no reason stated	13524 (0.6)	204 (1.0)	13320 (0.6)	
	Recommended, not administered, refused by patient, patient's family member or guardian	108560 (4.6)	680 (3.3)	107880 (4.6)	
	Hormone therapy recommended, unknown if administered	37580 (1.6)	443 (2.2)	37137 (1.6)	
	Unknown if recommended or administered	34828 (1.5)	349 (1.7)	34479 (1.5)	

Delay in surgery >60 days	Perform the treatment within 60 days	1795741 (76.0)	13829 (67.3)	1781912 (76.1)	<0.001
	Perform the treatment greater than 60 days	387115 (16.4)	4822 (23.5)	382293 (16.3)	
	Unknown	180771 (7.6)	1910 (9.3)	178861 (7.6)	
Delay in radiation >60 days	Perform the treatment within 60 days	215171 (9.1)	963 (4.7)	214208 (9.1)	<0.001
	Perform the treatment greater than 60 days	1097504 (46.4)	10115 (49.2)	1087389 (46.4)	
	Unknown	1050952 (44.5)	9483 (46.1)	1041469 (44.4)	
Delay in chemo >60 days	Perform the treatment within 60 days	388159 (16.4)	4342 (21.1)	383817 (16.4)	<0.001
	Perform the treatment greater than 60 days	359477 (15.2)	3546 (17.2)	355931 (15.2)	
	Unknown	1615991 (68.4)	12673 (61.6)	1603318 (68.4)	
Delay in surgery		44.62 (55.97)	56.19 (65.96)	44.52 (55.87)	<0.001
Delay in radiation		134.93 (86.44)	161.42 (93.79)	134.70 (86.34)	<0.001
Delay in chemotherapy		65.12 (53.48)	64.45 (69.23)	65.13 (53.29)	0.261

Supplementary Table S2. Treatment Receipt, Delays, and Surgical Characteristics Stratified by Stage at Diagnosis

		**Overall**	**South Asian**	**NHW**	**p-value**
**All Patients**		2363627	20561	2343066	
Surgery	Don't have the treatment	146566 (6.2)	1417 (6.9)	145149 (6.2)	<0.001
	Have the treatment	2196392 (92.9)	18863 (91.7)	2177529 (92.9)	
	Unknown	20669 (0.9)	281 (1.4)	20388 (0.9)	
Delay in surgery		44.62 (55.97)	56.19 (65.96)	44.52 (55.87)	<0.001
Surgical margins status	No residual tumor All margins are grossly and microscopically negative	2075372 (87.8)	17861 (86.9)	2057511 (87.8)	<0.001
	Residual Tumor	92460 (3.9)	731 (3.6)	91729 (3.9)	
	No primary site surgery, No surgical procedure of the primary site Diagnosed at autopsy	162110 (6.9)	1636 (8.0)	160474 (6.8)	
	Unknown or not applicable	33685 (1.4)	333 (1.6)	33352 (1.4)	
Surgical approach at this facility	Unknown	669624 (28.3)	3719 (18.1)	665905 (28.4)	<0.001
	No Surgical Procedure of Primary Site	400779 (17.0)	4060 (19.7)	396719 (16.9)	
	Surgery	1292613 (54.7)	12782 (62.1)	1279831 (54.6)	
	Open or approach unspecified	1280160 (54.2)	12632 (61.4)	1267528 (54.1)	
	Unknown whether surgery performed at this facility	611 (0.0)	0 (0.0)	611 (0.0)	
Radiation	Don't have the treatment	956250 (40.5)	8450 (41.1)	947800 (40.5)	<0.001
	Have the treatment	1333167 (56.4)	11376 (55.3)	1321791 (56.4)	
	Unknown	74210 (3.1)	735 (3.6)	73475 (3.1)	
Delay in radiation		134.93 (86.44)	161.42 (93.79)	134.70 (86.34)	<0.001

Chemotherapy	Don't have the treatment	1935466 (81.9)	15246 (74.2)	1920220 (82.0)	<0.001
	Have the treatment	396116 (16.8)	4999 (24.3)	391117 (16.7)	
	Unknown	32043 (1.4)	316 (1.5)	31727 (1.4)	
Delay in chemotherapy		65.12 (53.48)	64.45 (69.23)	65.13 (53.29)	0.261
Hormone	Don't have the treatment	830694 (35.1)	7287 (35.4)	823407 (35.1)	<0.001
	Have the treatment	1460525 (61.8)	12482 (60.7)	1448043 (61.8)	
	Unknown	72407 (3.1)	792 (3.9)	71615 (3.1)	
Hormone therapy at any CoC facility	Unknown	1 (0.0)	0 (0.0)	1 (0.0)	<0.001
	None	677433 (28.7)	6242 (30.4)	671191 (28.6)	
	Hormone therapy administered as first course therapy	1460525 (61.8)	12482 (60.7)	1448043 (61.8)	
	Not recommended/administered, contraindicated due to patient risk factors	28229 (1.2)	154 (0.7)	28075 (1.2)	
	Hormone therapy not administered, patient died prior to planned or recommended therapy	2948 (0.1)	7 (0.0)	2941 (0.1)	
	Recommended but not administered, no reason stated	13524 (0.6)	204 (1.0)	13320 (0.6)	
	Recommended, not administered, refused by patient, patient"s family member or guardian	108560 (4.6)	680 (3.3)	107880 (4.6)	
	Hormone therapy recommended, unknown if administered	37580 (1.6)	443 (2.2)	37137 (1.6)	
	Unknown if recommended or administered	34827 (1.5)	349 (1.7)	34478 (1.5)	
**Stage 1**		1091271	8237	1083034	
Surgery	No	20361 (1.9)	183 (2.2)	20178 (1.9)	<0.001
	Yes	1066287 (97.7)	7975 (96.8)	1058312 (97.7)	

	Unknown	4623 (0.4)	79 (1.0)	4544 (0.4)	
Delay in surgery		39.89 (43.74)	48.31 (51.02)	39.83 (43.68)	<0.001
Radiation	No	385087 (35.3)	2994 (36.3)	382093 (35.3)	0.009
	Yes	678786 (62.2)	5007 (60.8)	673779 (62.2)	
	Unknown	27398 (2.5)	236 (2.9)	27162 (2.5)	
Delay in radiation		111.91 (68.24)	134.34 (78.00)	111.74 (68.13)	<0.001
Chemo therapy	No	951421 (87.2)	6646 (80.7)	944775 (87.2)	<0.001
	Yes	125792 (11.5)	1471 (17.9)	124321 (11.5)	
	Unknown	14058 (1.3)	120 (1.5)	13938 (1.3)	
Delay in chemotherapy		72.65 (54.64)	74.71 (78.47)	72.63 (54.36)	0.069
Hormone	No	276396 (25.3)	1898 (23.0)	274498 (25.3)	<0.001
	Yes	785759 (72.0)	6067 (73.7)	779692 (72.0)	
	Unknown	29116 (2.7)	272 (3.3)	28844 (2.7)	
**Stage 2**		508235	4929	503306	
Surgery	No	18407 (3.6)	194 (3.9)	18213 (3.6)	<0.001
	Yes	485674 (95.6)	4669 (94.7)	481005 (95.6)	
	Unknown	4154 (0.8)	66 (1.3)	4088 (0.8)	
Delay in surgery		53.23 (66.32)	68.61 (78.94)	53.08 (66.17)	<0.001
Radiation	No	204356 (40.2)	1876 (38.1)	202480 (40.2)	0.001
	Yes	286443 (56.4)	2853 (57.9)	283590 (56.3)	

	Unknown	17436 (3.4)	200 (4.1)	17236 (3.4)	
Delay in radiation		185.60 (88.13)	208.96 (93.71)	185.36 (88.04)	<0.001
Chemo therapy	No	347283 (68.3)	2847 (57.8)	344436 (68.4)	<0.001
	Yes	155033 (30.5)	2020 (41.0)	153013 (30.4)	
	Unknown	5918 (1.2)	62 (1.3)	5856 (1.2)	
Delay in chemo		65.50 (47.62)	63.57 (69.05)	65.52 (47.33)	0.02
Hormone	No	162418 (32.0)	1691 (34.3)	160727 (31.9)	<0.001
	Yes	333939 (65.7)	3072 (62.3)	330867 (65.7)	
	Unknown	11878 (2.3)	166 (3.4)	11712 (2.3)	
**Stage 3**		175381	1823	173558	
Surgery	No	11950 (6.8)	126 (6.9)	11824 (6.8)	0.049
	Yes	161341 (92.0)	1664 (91.3)	159677 (92.0)	
	Unknown	2090 (1.2)	33 (1.8)	2057 (1.2)	
Delay in surgery		70.56 (82.63)	86.13 (96.53)	70.40 (82.46)	<0.001
Radiation	No	44884 (25.6)	415 (22.8)	44469 (25.6)	0.015
	Yes	123967 (70.7)	1331 (73.0)	122636 (70.7)	
	Unknown	6530 (3.7)	77 (4.2)	6453 (3.7)	
Delay in radiation		229.12 (74.09)	243.69 (66.63)	228.97 (74.15)	<0.001
Chemotherapy	No	102354 (58.4)	861 (47.2)	101493 (58.5)	<0.001
	Yes	71661 (40.9)	943 (51.7)	70718 (40.7)	
	Unknown	1366 (0.8)	19 (1.0)	1347 (0.8)	

Delay in chemotherapy		59.07 (47.60)	58.98 (59.26)	59.08 (47.45)	0.938
Endocrine Treatment	No	63879 (36.4)	727 (39.9)	63152 (36.4)	0.005
	Yes	107240 (61.1)	1047 (57.4)	106193 (61.2)	
	Unknown	4262 (2.4)	49 (2.7)	4213 (2.4)	
**Stage 4**		90365	793	89572	
Surgery	No	66421 (73.5)	578 (72.9)	65843 (73.5)	0.609
	Yes	21737 (24.1)	199 (25.1)	21538 (24.0)	
	Unknown	2207 (2.4)	16 (2.0)	2191 (2.4)	
Delay in surgery		81.23 (117.70)	102.25 (109.79)	81.04 (117.75)	0.007
Radiation	No	57514 (63.6)	515 (64.9)	56999 (63.6)	0.173
	Yes	29173 (32.3)	256 (32.3)	28917 (32.3)	
	Unknown	3678 (4.1)	22 (2.8)	3656 (4.1)	
Delay in radiation		99.93 (125.04)	136.54 (137.34)	99.62 (124.89)	<0.001
Chemotherapy	No	57457 (63.6)	392 (49.4)	57065 (63.7)	<0.001
	Yes	31711 (35.1)	386 (48.7)	31325 (35.0)	
	Unknown	1197 (1.3)	15 (1.9)	1182 (1.3)	
Delay in chemotherapy		46.99 (64.56)	49.17 (55.93)	46.96 (64.65)	0.436
Endocrine Treatment	No	39797 (44.0)	351 (44.3)	39446 (44.0)	0.075
	Yes	48638 (53.8)	416 (52.5)	48222 (53.8)	
	Unknown	1930 (2.1)	26 (3.3)	1904 (2.1)	

Supplementary Table S3. Breast Cancer Survival Outcomes by Stage and Ethnicity (Non-Hispanic White vs. South Asian Patients)

**All patients**	**NHW**	**South Asian**
Median (95% CI)	225.81 (224.56, NA)	NA (NA, NA)
5-year survival rate (95% CI)	0.87 (0.87, 0.87)	0.93 (0.92, 0.93)
10-year survival rate (95% CI)	0.76 (0.76, 0.76)	0.87 (0.86, 0.88)
15-year survival rate (95% CI)	0.64 (0.63, 0.64)	0.81 (0.8, 0.82)
Stage I		
Median (95% CI)	226.27 (225.81, NA)	NA (NA, NA)
5-year survival rate (95% CI)	0.93 (0.93, 0.93)	0.97 (0.97, 0.97)
10-year survival rate (95% CI)	0.82 (0.81, 0.82)	0.93 (0.92, 0.94)
15-year survival rate (95% CI)	0.68 (0.68, 0.68)	0.87 (0.85, 0.89)
Stage II		
Median (95% CI)	220.45 (218.55, 222.82)	NA (NA, NA)
5-year survival rate (95% CI)	0.86 (0.8, 0.86)	0.93 (0.92, 0.94)
10-year survival rate (95% CI)	0.73 (0.73, 0.73)	0.86 (0.85, 0.88)
15-year survival rate (95% CI)	0.61 (0.6, 0.61)	0.81 (0.79, 0.83)
Stage III		
Median (95% CI)	139.83 (138.18, 141.31)	NA (NA, NA)
5-year survival rate (95% CI)	0.71 (0.71, 0.71)	0.84 (0.82, 0.86)
10-year survival rate (95% CI)	0.54 (0.54, 0.54)	0.72 (0.69, 0.75)
15-year survival rate (95% CI)	0.43 (0.42, 0.43)	0.63 (0.59, 0.68)
Stage IV		
Median (95% CI)	30.59 (30.23, 30.98)	54.6 (47.11, 64.07)
5-year survival rate (95% CI)	0.3 (0.29, 0.3)	0.47 (0.43, 0.51)
10-year survival rate (95% CI)	0.14 (0.14, 0.15)	0.3 (0.25, 0.36)
15-year survival rate (95% CI)	0.09 (0.09, 0.09)	0.24 (0.18, 0.32)

Supplementary Table S4: Multivariable Cox Proportional Hazards Regression Analysis of Overall Survival in Breast Cancer Patients

For all patients		Hazard Ratio	Confidence interval 95% lower	Confidence interval 95% upper	p-value
Race	NHW	ref			
	South Asian	0.545	0.503	0.591	<0.0001
Age group	20-49	Ref			
	50-64	1.417	1.388	1.447	<0.0001
	65-74	1.911	1.864	1.961	<0.0001
	75+	4.478	4.364	4.596	<0.0001
Insurance status	Not Insured	Ref			
	Private Insurance	0.602	0.578	0.627	<0.0001
	Medicaid	0.996	0.952	1.042	0.861876
	Medicare	0.829	0.795	0.864	<0.0001
	Other Government	0.741	0.691	0.796	<0.0001
	Insurance Status Unknown	0.780	0.732	0.831	<0.0001
Median income quartiles	>= 17.6%	ref			
	10.9-17.5%	0.943	0.927	0.959	<0.0001
	6.3-10.8%	0.869	0.854	0.884	<0.0001
	< 6.3%	0.755	0.743	0.769	<0.0001
Urban rural residence	Metro	Ref			
	Rural	1.016	0.978	1.056	0.408159
	Urban	0.993	0.978	1.009	0.40931
Stage	Stage I	Ref			
	Stage II	1.747	1.724	1.770	<0.0001
	Stage III	3.763	3.699	3.827	<0.0001
	Stage IV	7.005	6.849	7.163	<0.0001
Grade	1	ref			
	2	1.170	1.152	1.188	<0.0001
	3	1.569	1.542	1.597	<0.0001

Subtype	HR-/HER2+	ref			
	HR+/HER2-	1.437	1.398	1.478	<0.0001
	HR+/HER2+	1.260	1.222	1.299	<0.0001
	Triple Negative	1.645	1.601	1.691	<0.0001
Charlson-Deyo Comorbidity Index	0	Ref			
	1	1.429	1.409	1.449	<0.0001
	2	1.954	1.909	2.000	<0.0001
	>= 3	2.583	2.500	2.668	<0.0001
Chemotherapy	No	Ref			
	Yes	1.033	1.016	1.051	0.000172
	Unknown	0.881	0.833	0.931	<0.0001
Radiation	No	Ref			
	Yes	0.694	0.657	0.735	<0.0001
	Unknown	0.94	0.912	0.969	<0.0001
Surgery	No	ref			
	Yes	0.432	0.413	0.451	<0.0001
	Unknown	0.623	0.585	0.663	<0.0001
Hormone	No	Ref			
	Yes	0.626	0.617	0.634	<0.0001
	Unknown	0.858	0.824	0.895	<0.0001
Delay in surgery >60 Days	No	ref			
	Yes	1.298	1.279	1.318	<0.0001
	Unknown	1.078	1.034	1.124	0.0004
Delay in radiation >60 Days	No	ref			
	Yes	0.891	0.873	0.910	<0.0001
	Unknown	0.834	0.786	0.883	<0.0001
Delay in chemotherapy >60 Days	No	ref			
	Yes	0.865	0.849	0.881	<0.0001
	Unknown	1.1897	1.1678	1.212	<0.0001
Facility type	Community Cancer Program	ref			

	Comprehensive Community Cancer Program	0.9141	0.8976	0.931	<0.0001
	Academic/Research Program	0.8081	0.7921	0.824	<0.0001
	Integrated Network Cancer Program	0.8864	0.8686	0.905	<0.0001

Supplementary Table S5: Multivariable Cox Proportional Hazards Regression for Overall Survival Among South Asian Breast Cancer Patients

South Asian only		Hazard Ratio	Confidence interval 95% lower	Confidence interval 95% upper	p-value
Age group	20-49	ref			
	50-64	1.22	1.07	1.39	0.003
	65-74	1.60	1.38	1.87	<0.0001
	75+	5.02	4.31	5.84	<0.0001
Insurance status	Not Insured	ref			
	Private Insurance	0.38	0.32	0.46	<0.0001
	Medicaid	0.83	0.67	1.04	0.11
	Medicare	0.97	0.78	1.18	0.71
	Other Government	0.38	0.18	0.81	0.01
	Insurance Status Unknown	0.67	0.48	0.95	0.02
Median income quartiles	>= 17.6%	ref			
	10.9-17.5%	1.19	0.89	1.57	0.23
	6.3-10.8%	1.19	0.92	1.55	0.19
	< 6.3%	0.97	0.76	1.23	0.78
Urban rural residence	Metro	ref			
	Rural	1.50	0.75	3.01	0.25
	Urban	0.98	0.63	1.50	0.91
Stage	Stage I	ref			
	Stage II	1.97	1.71	2.28	<0.0001
	Stage III	4.69	4.03	5.47	<0.0001
	Stage IV	19.96	17.14	23.25	<0.0001
Grade	1	ref			
	2	1.57	1.27	1.95	<0.0001
	3	2.55	2.07	3.15	<0.0001

Subtype	HR-/HER2+	ref			
	HR+/HER2-	0.81	0.62	1.06	0.13
	HR+/HER2+	1.02	0.75	1.39	0.91
	Triple Negative	1.74	1.30	2.32	<0.0001
Charlson-Deyo Comorbidity Index	0	ref			
	1	1.42	1.23	1.64	<0.0001
	2	2.61	1.94	3.51	<0.0001
	>= 3	4.21	2.85	6.21	<0.0001
Chemo therapy	None	ref			
	Yes	1.20	1.08	1.34	<0.0001
	Unknown	0.83	0.52	1.34	0.45
Radiation	None	ref			
	Yes	0.61	0.55	0.68	<0.0001
	Unknown	0.74	0.56	0.98	0.034699
Surgery	None	ref			
	Yes	0.11	0.09	0.12	<0.0001
	Unknown	0.32	0.21	0.49	<0.0001
Hormone	No	ref			
	Yes	0.49	0.44	0.55	<0.0001
	Unknown	0.44	0.31	0.62	<0.0001
Delay in surgery >60 Days	No	ref			
	Yes	1.59	1.40	1.81	<0.0001
	Unknown	6.64	5.88	7.49	<0.0001
Delay in radiation >60 Days	No	ref			
	Yes	0.57	0.46	0.71	<0.0001
	Unknown	0.96	0.78	1.20	0.73
Delay in chemo >60 Days	No	ref			
	Yes	0.56	0.48	0.64	<0.0001

	Unknown	0.63	0.57	0.71	<0.0001
Facility type	Community Cancer Program	ref			
	Comprehensive Community Cancer Program	0.88	0.71	1.11	0.28
	Academic/Research Program	0.75	0.60	0.94	0.012
	Integrated Network Cancer Program	0.96	0.76	1.22	0.74
